# The Genetic Backdrop of Hypogonadotropic Hypogonadism

**DOI:** 10.3390/ijms222413241

**Published:** 2021-12-08

**Authors:** Anna Szeliga, Michal Kunicki, Marzena Maciejewska-Jeske, Natalia Rzewuska, Anna Kostrzak, Blazej Meczekalski, Gregory Bala, Roman Smolarczyk, Eli Y. Adashi

**Affiliations:** 1 Department of Gynecological Endocrinology, Poznan University of Medical Sciences, 60-535 Poznan, Poland; annamaria.szeliga@gmail.com (A.S.); marzena@jeske.pl (M.M.-J.); ankos30@op.pl (A.K.); 2 INVICTA Fertility and Reproductive Center, 00-019 Warsaw, Poland; mkunicki@wum.edu.pl; 3 Department of Gynecological Endocrinology, Medical University of Warsaw, 00-315 Warsaw, Poland; bnrzewuska@gmail.com (N.R.); rsmolarczyk@poczta.onet.pl (R.S.); 4 Appletree Medical Group, Ottawa, ON K1R 5C1, Canada; greg.bala1@gmail.com; 5 Warren Alpert Medical School, Brown University, 272 George St., Providence, RI 02906, USA; eli_adashi@brown.edu

**Keywords:** pituitary amenorhhea, GPR54, DAX-1, FGFR-1, HESX1

## Abstract

The pituitary is an organ of dual provenance: the anterior lobe is epithelial in origin, whereas the posterior lobe derives from the neural ectoderm. The pituitary gland is a pivotal element of the axis regulating reproductive function in mammals. It collects signals from the hypothalamus, and by secreting gonadotropins (FSH and LH) it stimulates the ovary into cyclic activity resulting in a menstrual cycle and in ovulation. Pituitary organogenesis is comprised of three main stages controlled by different signaling molecules: first, the initiation of pituitary organogenesis and subsequent formation of Rathke’s pouch; second, the migration of Rathke’s pouch cells and their proliferation; and third, lineage determination and cellular differentiation. Any disruption of this sequence, e.g., gene mutation, can lead to numerous developmental disorders. Gene mutations contributing to disordered pituitary development can themselves be classified: mutations affecting transcriptional determinants of pituitary development, mutations related to gonadotropin deficiency, mutations concerning the beta subunit of FSH and LH, and mutations in the DAX-1 gene as a cause of adrenal hypoplasia and disturbed responsiveness of the pituitary to GnRH. All these mutations lead to disruption in the hypothalamic–pituitary–ovarian axis and contribute to the development of primary amenorrhea.

## 1. The Pituitary and Its Role in Reproduction

The pituitary is an organ of dual provenance: the anterior lobe is epithelial in origin, whereas the posterior lobe derives from the neural ectoderm. Functionally, it is closely related to the hypothalamus. From the anterior lobe, the pituitary secretes growth hormone (GH), prolactin (PRL), adrenocorticotropic hormone (ACTH), thyrotropic hormone (TSH), luteinizing hormone (LH), and follicle stimulating hormone (FSH). The posterior pituitary stores oxytocin and vasopressin (ADH), which are synthesized in the anterior hypothalamus (paraventricular nucleus and supraoptic nucleus).

The pituitary gland is a pivotal element of the axis regulating reproductive function in mammals. It collects signals from the hypothalamus, and by secreting gonadotropins (FSH and LH), it stimulates the ovary into cyclic activity, resulting in a menstrual cycle and ovulation.

Proper temporal and spatial coordination of the expression of transcription factors in both structural subsections of the pituitary is pivotal to proper pituitary formation and subsequent differentiation of hormone-producing cells [1].

Pituitary organogenesis is comprised of three main stages controlled by different signaling molecules: first, the initiation of pituitary organogenesis and the subsequent formation of Rathke’s pouch; second, the migration of Rathke’s pouch cells and their proliferation; and third, lineage determination and cellular differentiation. Any disruption of this regulation, e.g., gene mutation, can lead to numerous developmental disorders [1].

Gene mutations contributing to the disruption of pituitary development can themselves be classified: mutations affecting transcriptional determinants of pituitary development, mutations causal to gonadotropin deficiency, mutations affecting the beta subunit of FSH and LH, and mutations in the DAX-1 gene as a cause of adrenal hypoplasia and of disturbed responsiveness of the pituitary to GnRH (Table 1). All these mutations lead to disruption in the hypothalamic–pituitary–ovarian axis and thereby contribute to development of primary amenorrhea. Primary amenorrhea is defined as the absence of menses by age 15 years in the presence of normal growth and secondary sexual characteristics. However, at age 13 years, if no menses have occurred and there is a complete absence of secondary sexual characteristics such as breast development, an evaluation should be initiated into primary amenorrhea. Primary amenorrhea is usually the result of a genetic or anatomical abnormality. The most common cause of primary amenorrhea (including Turner syndrome) is gonadal dysgenesis; this is the cause in about 43 percent of primary amenorrhea cases. Genetic causes of pituitary amenorrhea account for about 5% of cases. As this is a commonly underestimated and often neglected problem, we have decided to summarize the most common gene mutations contributing to this problem.

## 2. Mutations of the Beta Subunits of FSH and LH

### 2.1. Mutations of the Beta Subunit of FSH

Both pituitary gonadotropins (follicle-stimulating hormone (FSH) and Luteinizing Hormone (LH)) are composed of 2 subunits (alpha and beta). The alpha subunit is common in both gonadotropins, while the beta subunit is distinct [2]. FSH-β is a gene encoding the FSH-β subunit; it is comprised of 3 exons located at chromosome 11.14.1 and composed of 129 amino acids [2].

Accurate gonadotropin subunit structure determines proper gonadotropin functionality. To date, no mutations of the α subunit have been reported. Mutations of FSH-β and LH-β subunits, however, have been associated with primary amenorrhea and delayed puberty [3].

In 1972, Rabin et al. [4] reported on the case of an Israeli women who presented with primary amenorrhea, which was later discovered to be caused by an isolated FSH deficiency. After seven years, a follow-up assessment of this same patient demonstrated that treatment with gonadotropins had successfully induced ovulation, which ultimately resulted in pregnancy [4].

Mutations in the FSH-β subunit have been reported both in females and males, with a higher case incidence in females. In 1993, an FSH-β subunit mutation was identified by Matthews et al. [3] as a homozygous 2-bp deletion in codon 61 (Val 61) of the FSH-β gene. This patient presented with primary amenorrhea and infertility [3]. In 1997, Layman et al. [5] uncovered an FSH-β subunit mutation in a female patient who presented with a similar phenotype.

The typical presenting phenotype of a patient with an FSH-β subunit mutation is primary amenorrhea and complete absence of pubertal development [5]. Several case reports, however, have also observed partial absence of pubertal development [6]. Complete inactivating mutations of FSH-β are a very rare autosomal recessive disorder and so far have been identified in only 12 patients (6 females and 6 males).

Misgar et al. [7] reported on an FSH-β subunit mutation present in 2 sisters. Molecular analysis revealed one nonsense mutation (c.343C>T:p Arg115Stop) in exon 3. Both sisters presented with undetectable serum FSH and estradiol, high serum LH, primary amenorrhea, and the absence of breast development.

Zhu et al. [8] identified a novel FSH-β subunit mutation (mutation Arg 97X) in a 29-year-old women suffering from primary amenorrhea, impaired pubertal development, and infertility. Similarly to the case described by Misgar, this patient also presented with an undetectable serum FSH, very low serum estradiol, and high serum LH levels.

One of the most concerning medical problems in this group of patients is infertility. According to Schoot et al. [9], the best therapeutic approach in a patient with FSH-β mutation is therapeutic FSH replacement combined with assisted reproductive technology (ART). Documenting an accurate genetic background and clinical phenotype in patients with FSH-β mutation is helpful in understanding the basis for this complicated abarrency of female reproduction.

### 2.2. Mutations of the Beta Bubunits of LH

The gene coding for the human LH-β subunit (*LHB*) is located on chromosome 19q13.3 and includes 5 non-coding pseudogenes. It is responsible for encoding a peptide, which is composed of 121 amino acids [10].

The first case of delayed puberty due to an LH gene mutation was described in 1992. The 17-year-old male proband was homozygous for a single base substitution in the LH-β subunit gene [11].

The first documented case of a female patient with LH-β subunit mutation was described in 2007. Lofrano-Porto et al. [12] reported on a consanguineous family (2 males and 1 female) in which all 3 patients displayed hypogonadotropic hypogonadism (HH) due to an LH-β subunit mutation. It is interesting to note that the female patient in question presented with normal pubertal development but developed secondary amenorrhea and subsequent infertility. To date, all female patients with reported *LHB* gene mutations are sisters of 46,XY probands, having a corresponding mutation in the LH protein [13].

These female patients are characterized by female external genitalia, and exhibit spontaneous breast and pubic hair development at puberty. They feature normal or late menarche and infertility. They also exhibit a typical hormonal profile comprising low serum LH and normal FSH levels. Serum levels of estradiol are low and do not reach ovulatory levels, thereby causing anovulation [13]. Treatment of women with *LHB* gene mutations includes the use of exogenous hCG and LH [14].

In a similar effort as with FSH-β subunit mutations, these studies provide insight and a better understanding of the causes of amenorrhea, reproductive dysfunction, and infertility.

## 3. Gene Mutations Affecting Transcriptional Determinants of Pituitary Development

Primary amenorrhea may occur as a result of a mutation in the genes encoding transcription factors, which are involved in cellular proliferation and differentiation of the pituitary gland. *HESX1, GLI2*, and *SOX3* mutations have been linked to hypopituitarism, and mutations in *SOX2, LHX3, LHX4*, and *PROP1* may cause gonadotropin deficiency [15].

Alterations in these genes will often also be expressed as clinical findings that aid in the diagnosis. For example, mutations in *SOX2* may result in anophthalmia, and mutations in *LHX3* have been associated with a rotationally limited cervical spine [15].

### 3.1. HESX1

The paired-like homeobox gene, *HESX1* (homeobox expressed in embryonic stem cells), is one of the earliest markers of pituitary development [16]. This gene encodes a conserved homeobox protein that functions as a transcriptional repressor in forebrain and pituitary gland development [17]. It is an early marker of Rathke’s pouch. Its downregulation is essential for cell differentiation [18].

Mutations in the *HESX1* gene follow autosomal dominant or autosomal recessive inheritance [19]. The *HESX1* gene is necessary for the proper development of the anterior hypophysial structures such as the olfactory placodes and the pituitary gland. It also contributes to the proper development of the forebrain and the eyes [17]. Therefore, mutations of *HESX1* are associated with isolated GH deficiency (IGHD) and combined pituitary hormone deficiency (CPHD) [19,20]. Additionally, *HESX1* mutations can cause hypoplasia or even aplasia of the anterior pituitary with or without hypoplasia of the corpus callosum [19]. *HESX1* mutations contribute to development of septo-optic dysplasia (SOD), a condition that can be diagnosed with the presence of two or more features of the classical triad: (i) optic nerve hypoplasia, (ii) pituitary hormone abnormalities, and (iii) midline brain defects, including agenesis of the septum pellucidum and/or corpus callosum [21].

### 3.2. GLI2

*GLI2* (GLI Family Zinc Finger 2) codes for a protein belonging to the C2H2-type zinc finger subclass of the GLI protein family. Members belonging to this subclass are characterized as transcription factors that bind DNA through zinc finger motifs [22].

GLI zinc finger proteins are mediators of Sonic hedgehog (SHH) signaling and considered potent oncogenes involved in embryonal carcinoma cell development. The SHH signaling pathway, which activates target genes under the control of the GLI family of transcription factors, is the best studied cause of holoprosencephaly [22,23].

In humans, the most common *GLI2* mutations have been associated with holoprosencephaly. Most cases (85%) feature holoprosencephaly, cleft palate, or polydactyly. In certain *GLI2* variants, IGHD (13%), hypopituitarism with unspecified hormone deficiencies (6%), and HH (2%) have also been associated [23].

*GLI2* sequence abnormalities have also been shown to cause eye, ocular orbit, nose, first branchial arch, and a varying degree of gyral development deformity. The facial phenotype resulting from first branchial arch malformation is most likely related to the extended action of the SHH signaling pathway [24].

Mutations relating to the SHH pathway are associated with several phenotypes: Greig cephalopolysyndactyly syndrome, Pallister–Hall syndrome, preaxial polydactyly type IV, and postaxial polydactyly types A1 and B [22]. Additionally, when associated with *GLI2* they can also include Culler–Jones Syndrome [25].

### 3.3. SOX3

*SOX3* (SRY-Box Transcription Factor 3) is an X-linked transcription factor involved in early-stage pituitary development [26]. This gene encodes a member of the *SOX* (SRY-related HMG-box) family. It keeps neural cells undifferentiated by counteracting the activity of proneural signaling proteins and suppresses neuronal differentiation [27]. *SOX3* is an important factor in normal hypothalamo-pituitary development, and its disruption is associated with panhypopituitarism, craniofacial abnormalities, mental retardation, and midline defects [26,27,28,29]. Mutations in this gene have also been associated with X-linked growth hormone deficiency [27]. In most cases, such mutations cause the retention of an ectopic/undescended posterior pituitary [26]. It is suggested that normal development of the hypothalamus and pituitary gland regions is critically dependent on a *SOX3* margin of concentration. Duplication causing overdosage or underdosage caused by polyalanine tract expansion can lead to hypopituitarism [30].

### 3.4. FGFR-1

The *FGFR1* gene codes for fibroblast growth factor receptor 1, a member of the fibroblast growth factor receptor (FGFR) family. Amino acid sequences are highly conserved between members of this family and largely unchanged throughout evolution.

Mutations in this gene have been associated with Pfeiffer syndrome, Jackson–Weiss syndrome, Antley–Bixler syndrome, osteoglophonic dysplasia, and autosomal dominant Kallmann syndrome. Chromosomal aberrations involving this gene are associated with stem cell myeloproliferative disorder and stem cell leukemia lymphoma syndrome.

*FGFR1* controls normal mesoderm patterning, embryonic development, differentiation specification, migration, cell proliferation, and survival of GnRH-secreting neurons. It is required for correct axial organization during embryonic development, normal skeletogenesis, and normal development of the gonadotropin-releasing hormone (GnRH) neuronal system [31]. In humans, mutations in these genes lead to severe congenital GnRH deficiency: idiopathic hypogonadotropic hypogonadism (IHH) [32]. *FGFR1* mutations can cause IHH with normal olfaction, and this normosmic IHH can present with GnRH deficiency ranging from completely absent puberty to partial or even delayed puberty. Thus, heterozygous *FGFR1* loss of function mutations may cause isolated defects in GnRH neuronal migration without necessarily affecting olfactory bulb development, and vice versa [33].

Patients who have hypothalamic amenorrhea with *FGFR1* mutation have abnormal patterns of endogenous GnRH-induced LH secretion. It is suspected that decreased *FGFR1* signaling leads to a partially compromised GnRH neuronal network owing to a smaller-than-normal number of GnRH-producing cells having successfully completed development [32].

### 3.5. GPR 54

The human *GPR54* gene (*KISS1R; KISS1* Receptor) is a protein-coding gene [34]. Since its discovery in 2001, kisspeptin has been known as the natural ligand of GPR54. As a consequence, GPR54 is referred to as the kisspeptin receptor (KISS1R). [35]

The *GPR54* gene has five exons, four introns, and is approximately 3.5 kb in size. It encodes a seven-transmembrane receptor with 398 amino acids [36]. The protein encoded by this gene is a galanin-like G protein-coupled receptor [34]. The ligand for GPR54 is encoded by the *KISS1* gene, which produces a 54-amino-acid peptide (kisspeptin-54) that can be cleaved into shorter peptides (kisspeptins 14, 13 and 10) that have similar activity [37].

Diseases associated with *KISS1R* mutations include both activating and inactivating mutations. Activating mutations cause hypersecretion of kisspeptin, which, in turn, leads to increased pulsatile GnRH secretion and hyperstimulation of the hypothalamic–pituitary–ovarian axis. This can contribute to the development of central precocious puberty (CPP). On the other hand, inactivating mutations cause a lack of kisspeptin action and thus prevent the activation of GnRH neurons, which leads to isolated hypogonadotropic hypogonadism (IHH) with or without anosmia [34,38,39,40,41,42].

## 4. Gene Mutations Related to Gonadotropin Deficiency

Gonadotropin deficiency (GD) is a disorder characterized by impaired production of gonadotropins, including insufficient secretion of LH (luteinizing hormone) and FSH (follicle-stimulating hormone). It can present in infancy, adolescence, or adulthood [43]. Gonadotropin deficiency may be isolated or part of an extended anterior pituitary hormone deficiency (multiple pituitary hormone deficiency—MPHD). The deficiency in one or more pituitary hormones results from a mutation of transcription factors related to pituitary development [43,44].

*SOX2* (SRY-Box Transcription Factor 2) is a member of the SOXB1 family of transcription factors, a group that is highly related to *SRY* (sex-determining region Y). *SOX2* is a transcription factor and marker of stem cells involved in pituitary gland development. *SOX2* expression persists throughout the development of the anterior pituitary gland and is also found in Rathke’s pouch [45]. Mutations in the *SOX2* gene may cause numerous malformations, including of the eyes, gastrointestinal, and genitourinary tract [46]. The presenting phenotype in these mutations typically includes anterior pituitary hypoplasia as reveled by MRI scan, and hypogonadotropic hypogonadism [44]. *SOX2* mutation is also associated with hypopituitarism. The latter is correlated with occular abnormalities.

Selective gonadotropin deficiency as a result of *SOX2* mutation, which spares other hormones of the hypothalamic-pituitary axis, has been described in the literature. This may indicate uneven *SOX2* expression in the hypothalamus. Patients who present with this mutation and associated ocular abnormalities are at high risk of gonadotropin deficiency and therefore require appropriate workup and treatment for sexual deficiency [47].

*PROP1* (homeobox protein prophet of PIT1) is the most frequently mutated gene known to cause pituitary hormone dysfunction [23]. *PROP1* is crucial in the development of most cells in the anterior pituitary gland. Therefore, mutation can result in a deficiency of all the anterior pituitary hormones [44]. Patients with a *PROP1* mutation are most often initially diagnosed with short stature due to growth hormone deficiency. This mutation is known to cause decreased TSH and PRL levels and can exhibit a shortage of LH and FSH as well [23]. Development of a *PROP1* mutation early on in life leads to gonadotropin deficiency, which in turn leads to cryptorchidism, impaired sexual maturation, and infertility.

At the onset of puberty, many patients are deficient in LH and FSH and do not develop secondary sexual characteristics. A loss of gonadotropin secretion may also develop in adulthood. *PROP1* mutation is more commonly observed in familial cases and among certain ethnic groups than it is in isolated or sporadic cases [44]. A *PROP1* mutation should be considered when a patient is observed to have GH, TSH, prolactin, and gonadotropins deficiency, especially in the absence of overt pituitary or posterior pituitary lesions on MRI, or in the case of intracranial pseudo-tumor [46].

*LHX3* and *LHX4* are transcription factors and members of the LIM-homeodomain family. *LHX3* and *LHX4* collectively regulate the proliferation and differentiation of pituitary-specific cell lines [48]. *LHX3* and *LHX4* are involved in regulating the genetic cascade necessary for the formation of Rathke’s pouch and the development of the pituitary gland [44]. *LHX3* and *LHX4* are variants of genes expressed early-on in pituitary development [49].

*LHX3* is crucial in the formation of spinal cord neurons [23], whereas its mutations have a presentation similar to that of a *PROP1* mutation. It is characterized by GH and TSH reduction along with PRL, gonadotropins, and ACTH deficiency [44]. An *LHX3* mutation should be suspected in patients with a decreased complement of anterior pituitary hormones in addition to symptoms such as limited neck rotation and sensorineural deafness [23,46]. In the case of heterozygous *LHX3* mutation, mild combined pituitary hormone deficiencies may occur [44].

*LHX4* mutations can cause isolated growth hormone deficiency or combined pituitary hormone deficiency (CPHD) [50]. *LHX4* mutations in familial cases of CPHD are autosomal-dominant with incomplete penetration. All mutations of *LHX4* known to date are heterozygous [51]. CPHD is associated with an ectopic posterior pituitary, anterior pituitary hypoplasia, a small sella turcica, and the possibility of Chiari malformation [44,46]. Heterozygous mutations manifest as decreased GH and LH, TSH, FSH, or ACTH [44].

## 5. Gene Mutations of DAX-1 and SF-1 as a Cause of Adrenal Hypoplasia and Disturbed Responsiveness of the Pituitary (LH) to GnRH

*DAX1* (dosage-sensitive sex reversal, adrenal hypoplasia critical region, on chromosome X, gene 1), also known as *NROB1* (nuclear receptor subfamily 0, group B, member 1), is a nuclear receptor which is involved in gonadal and adrenal development. It is expressed in the hypothalamus, pituitary, gonads, adrenals, and urogenital ridge [52,53]. Humans with *DAX1* mutation develop adrenal hypoplasia congenita (AHC), an X-linked condition. The mutations and deletions of *DAX1* and its association with X–linked adrenal hypoplasia were first described in 1994 [54].

The main features of AHC include primary adrenal insufficiency (PAI), and hypogonadotropic hypogonadism (HH), which leads to impaired fertility. The disease primarily affects boys, although mutations have been described in girls and women as a result of skewed X-inactivation. Most cases in girls are asymptomatic [55]. PAI is the main clinical presentation observed in male patients. Epidemiological data show more than half of males with PAI possess a mutation in the *DAX1* gene. These affected males typically present in infancy with adrenal failure but later fail to undergo puberty because of hypogonadotropic hypogonadism [56].

It is important to remember that aldosterone synthase deficiency, mineralocorticoid insufficiency leading to salt loss, or familial glucocorticoid deficiency can also feature as a presenting condition. The onset and severity of the clinical presentation will differ depending on the mutation and the type thereof [57].

The initial presentation of adrenal insufficiency will often manifest in the first few months of life. An affected infant will present with signs of both mineralocorticoid and glucocorticoid insufficiency leading to salt-loss and adrenal crisis. Vomiting, hyperpigmentation, vascular collapse, convulsions, and sudden death may develop if the condition is left untreated. Laboratory testing will reveal hyperkalemia along with hyponatremia and hypoglycemia. Moreover, low circulating cortisol and aldosterone levels, increased plasma renin activity, and elevate adrenocorticotropic hormone (ACTH) are often present as well [57].

It is important to note that despite possessing a *DAX-1* gene mutation, basal cortisol levels can remain within the normal range. Cortisol levels within the reference range do not exclude the presence of disease. Adrenal insufficiency is assumed to be a result of the impaired process of cellular transition from the fetal to the adult zone during development. In such cases, a differential diagnosis should include conditions connected with adrenal insufficiency such as congenital adrenal hyperplasia (most commonly 21-hydroxylase deficiency) and X-linked adrenoleukodystrophy. Rarely, the onset of PAI occurs in young adulthood [58]. In these cases, male patients present with signs of hypogonadotropic hypogonadism and delayed puberty. The latter is a feature of X-linked AHC. It is important to collect a thorough and detailed family history, including any potential signs of adrenal insufficiency such as unintentional weight loss, fatigue, and hyperpigmentation, among others. Puberty may be incomplete or delayed, or patients may have a complete absence of pubertal development altogether [59]. Hormonal findings usually reveal low serum gonadotropin levels and low serum testosterone. The pituitary does not respond when stimulated with GnRH pulses. Interestingly, gonadotropin-independent precocious puberty has been described in the literature wherein patients presented with the presence of pubic hair [60]. The adult patient will often suffer from azoospermia, while cryptorchidism (both uni- or bilateral) is the result of exposure to low levels of gonadotropins in utero. Treatment depends largely on the patients age at diagnosis and the attendant clinical manifestations. Infants with PAI should be treated parenterally with hydrocortisone as per standard guidelines for adrenal insufficiency. Both hydrocortisone and fludrocortisone can be used.

Initiation of pubertal development cannot be achieved through the use of pulsatile gonadoliberin (GnRH) owing to the pituitary’s unresponsiveness to GnRH caused by genetic mutation. Thus, puberty and virilization are achieved either by the injection of human chorionic gonadotropin (hCG), which stimulates testosterone production, or by initiating testosterone replacement therapy, which is often more patient-friendly and less costly [61,62].

Treatment with gonadotropins, in men affected by this mutation, is ineffective in stimulating spermatogenesis. Thus, azoospermia has been found after even long-term treatment. However, Frapsauce et al. reported that after 20 months treatment few spermatoza were obtained in testis biopsy. In that case, the live birth a boy was reported after testicular sperm extraction and intracytoplasmatic sperm injection (TESE-ICSI) [62].

Another factor to consider is Steroidogenic factor 1 (*SF-1*, also known as Ad4BP, encoded by *NR5A1*), which was recognized in the 1990s as a valid regulator of steroidogenic enzymes [63,64]. *SF-1* is coexpressed with *DAX-1* [65]. It is an orphan nuclear receptor that regulates transcriptions of many genes related to adrenal and gonadal development as well as the function of steroidogenic cells in these organs [63,66]. *SF-1* and *DAX-1* mutations impair the synthesis and function of adrenal steroids and contribute to the development of adrenal and gonadal clinical conditions. *DAX-1* and *SF-1* can inter-react to regulate gene transcription [64,67]. Ad4BP plays a key role in reproduction and adrenal function by affecting every level of the hypothalamic-pituitary-gonadal axis [56]. *SF-1* is also responsible for the control of genes in the steroid pathway such as LH-β, FSH-β, and steroid hydroxylase [65]. Changes in *SF-1* activity can contribute to a variety of disease states, mainly male factor infertility, primary ovarian insufficiency, and bilateral anorchia [63,64]. This mutation has also been described in a patient with adrenal failure [56]. It seems that changes in *SF-1* are most likely to cause sporadic primary adrenal failure, and significant underdevelopment of androgenization if they occur in 46,XY individuals. There is a hypothesis that suggests that changes in the *SF-1* gene may predispose one to late-onset adrenal insufficiency. Whereas the pathomechanism of Ad4BP mutation remains unknown, changes in *SF-1* lead to specific phenotypical traits. The final clinical picture of this mutation, however, is influenced by external factors, including environmental ones. Additionally, heterozygous *NR5A1* mutations are a relatively frequent finding in 46,XY disorders of sex development (46,XY DSD) presenting without adrenal insufficiency.

To summarize, *SF-1* is an important factor in the reproduction and function of the adrenal glands. Some of the diseases caused by changes in *SF-1* appear to be sporadic, such as adrenal dysfunction, whereas others occur with higher frequency, as in the case of primary ovarian insufficiency and male factor infertility [63].

## 6. IMAGe Syndrome

As an acronym for the most important components of this disorder, IMAGe is a syndrome comprised of intrauterine growth restriction, metaphyseal dysplasia, adrenal hypoplasia congenita, HH, and genital abnormalities [68]. IMAGe is a multisystem disease with potentially life-threatening complications such as adrenal insufficiency [68,69,70]. Many familial and isolated syndromes of IMAGe have been described [71]. The diagnosis is established based on clinical presentation and/or detection of the heterozygous CDKC1C pathogenic variant of proliferating cell nuclear antigen (PCNA) [70]. Therapeutic management includes addressing clinical manifestations of the syndrome, i.e., treatment of adrenal insufficiency, administration of growth hormone, surgical operations on cryptorchidism and hypospadias, and orthopedic interventions [69]. Typically, adrenal hypoplasia congenita (AHC) develops as a result of an isolated DAX1 mutation. As one of the components of IMAGe syndrome, and unlike its conventional presentation, AHC of IMAGe syndrome is not caused by a DAX1 nor an *SF-1* mutation [71].

## 7. Conclusions

In this review, we cover the genetic causes of primary amenorrhea. The mutations that can lead to disruption of the hypothalamic–pituitary–ovarian axis contribute to the development of idiopathic hypogonadotropic hypogonadism. In certain cases, additional sequalae such as adrenal insufficiency feature as well. We should be aware that in some cases, mutations may be familial. There remains extensive room for continued study into the genetic causes of amenorrhea and to further our understanding of the many different (e.g., environmental) factors that influence the phenotypic heterogeneity of this condition.

## Figures and Tables

**Table 1:** Summary of mutations contributing to hypogonadotropic hypogonadism.

Mutations of the Beta Subunits of FSH and LH		
FSH-α; LH-α	No mutations of α subunit have been reported.	
FSH-β; LH-β	Primary amenorrhea and delayed puberty, infertility, and hypogonadotropic hypogonadism.	
Gene mutations affecting transcriptional determinants of pituitary development		
HESX1	Necessary for the development of the olfactory placodes and the pituitary gland.	
	Associated with:	hypoplasia or aplasia of the anterior pituitary
		septo-optic dysplasia
		combined pituitary hormone deficiency
		isolated GH deficiency
*GLI2*	Associated with:	holoprosencephaly
		polydactyly
		cleft palate
		hypogonadotropic hypogonadism
		hypopituitarism with unspecified hormone deficiencies
		isolated GH deficiency
		eye, ocular orbit, nose, first branchial arch, and a varying degree of gyral development deformity
*SOX3*	Associated with:	panhypopituitarism
		craniofacial abnormalities
		mental retardation
		midline defects
		X-linked growth hormone deficiency
*FGFR-1*	Associated with:	Pfeiffer syndrome
		Jackson–Weiss syndrome
		Antley–Bixler syndrome
		osteoglophonic dysplasia
		autosomal dominant Kallmann syndrome
		isolated hypogonadotropic hypogonadism with normal olfaction
*GPR 54*	Associated with:	activating mutations: central precocious puberty
		inactivating mutations: isolated hypogonadotropic hypogonadism with or without anosmia
Gene mutations related to gonadotropin deficiency		
SOX2	Associated with:	malformations including of the eyes, gastrointestinal and genitourinary tract
		hypogonadotropic hypogonadism
		hypopituitarism
*PROP1*	Associated with:	deficiency of all the anterior pituitary hormones
*LHX3*	Associated with:	presentation similar to that of *PROP1* mutations
*LHX4*	Associated with:	isolated growth hormone deficiency or combined pituitary hormone deficiency
Gene mutations of *DAX-1* and *SF-1* as a cause of adrenal hypoplasia and disturbed responsiveness of the pituitary (LH) to GnRH		
*DAX1 and* *SF-1*	Involved in gonadal and adrenal development	
	Associated with:	adrenal hypoplasia congenita
		primary adrenal insufficiency
		hypogonadotropic hypogonadism
		impaired fertility
